# Early maladaptive schemas as common and specific predictors of skin picking subtypes

**DOI:** 10.1186/s40359-020-0392-y

**Published:** 2020-03-19

**Authors:** Andrea Pozza, Umberto Albert, Davide Dèttore

**Affiliations:** 1 Department of Medical Sciences, Surgery and Neurosciences, University of Siena, Viale Bracci 16, 53100 Siena, Italy; 2 Department of Medicine, Surgery and Health Sciences, University of Trieste, via de Pastrovic 4, 34128 Trieste, Italy; 3 Department of Health Sciences, University of Florence, via di San Salvi 12 - Building 26, 50135 Florence, Italy

**Keywords:** Skin picking, Dermatillomania, Schema therapy, Personality, Body focused repetitive behaviour, Emotion regulation

## Abstract

**Background:**

Three distinct subtypes of Skin Picking (SP) have been identified in previous research: Focused, Automatic and Mixed. Early Maladaptive Schemas (EMS) were not investigated across the subtypes. Understanding which EMS are associated with the subtypes might suggest the evaluation of Schema Therapy for SP and guide clinicians using it according to subtypes. The current study explored the relationship between EMS and SP subtypes in community adults.

**Methods:**

Five hundred ninety-six adults [mean age = 35.23 years, 66% females] self-reporting SP behaviours completed the Milwaukee Inventory for Dimensions of Adult Skin Picking and the Young Schema Questionnaire-Long form third version (YSQ-L3).

**Results:**

Higher Dependence/Incompetence EMS was a common predictor of both Focused and Automatic subtypes, while lower Emotional Deprivation EMS and younger age predicted all three subtypes. Higher Approval/Recognition Seeking, Mistrust/Abuse and Failure to Achieve were specific predictors of Automatic, Focused and Mixed subtypes, respectively. Lower Social Isolation/Alienation and Enmeshment/Undeveloped Self were specific predictors of Focused subtype. Male gender was a specific predictor of Mixed subtype.

**Conclusions:**

The assessment and psychological treatment of individuals with SP behaviour may focus on specific EMS. Future longitudinal studies using clinical samples may clarify this association.

## Background

### Skin picking subtypes

Skin Picking (SP) consists of repetitive picking behaviours associated with distress and often social avoidance, consequence of skin damage caused by picking [1]. Individuals pick small irregularities or skin lesions (scars, pimples or scabs), resulting from previous picks, in the most accessible areas, such as face, arms/hands/legs [2]. Prevalence ranges between 1.4 and 5.4% in the community [1] with women and younger people reporting this behaviour more frequently [3].

Different subtypes with distinct functional characteristics were identified. Arnold and colleagues [4] identified three subtypes: (1) Compulsive SP, performed in full awareness to cope with negative emotions; (2) Impulsive/Automatic SP, performed with minimal awareness; (3) a Mixed subtype sharing features of both. In non-clinical individuals, Walther and colleagues [5] identified two subtypes: an Automatic one, that tends to occur outside of one’s awareness, including situations where the individual picks his/her skin while engaged in a sedentary activity (e.g., reading/watching television); a Focused subtype, performed to cope with negative feelings or to correct perceived imperfections. These characteristics of the SP subtypes have been confirmed in further studies where the Automatic one correlated with measures of emotional clarity and the Focused one with measures of emotion regulation difficulties [6]. In non-clinical samples Pozza and colleagues [3] reported three subtypes, including an Automatic, a Focused and a Mixed one.

The distinction between subtypes has implications for practice. Focused SP would be associated with emotion regulation deficits, whereas the Automatic subtype would be related to poorer awareness of picking [7]. Various emotion regulation difficulties, including impulsiveness, were associated with Focused/Compulsive subtype [8]. It might be expected that Focused SP benefits from treatment increasing emotion regulation, whereas Automatic SP benefits from treatment increasing awareness of picking behaviour and the individual’s capacity to stop it [7]. However, research provided mixed findings about the specificity of emotion regulation deficits to Focused subtype, as they correlated also with the Automatic one [9].

### Personality constructs and early maladaptive schemas in SP

Little research explored personality dimensions across subtypes. In non-clinical/clinical samples avoidant personality traits predicted Automatic SP, borderline traits predicted both Automatic and Focused SP and sadistic ones predicted Mixed SP [10].

A construct related to personality and interpersonal processes are Early Maladaptive Schemas (EMS), defined as “*a broad, pervasive theme or pattern, comprised of memories, emotions, cognitions, and bodily sensations, regarding oneself and one’s relationships with others, developed during childhood or adolescence, elaborated throughout one’s lifetime and dysfunctional to a significant degree*” [11]. EMS are trait-like features which develop through interactions between temperament and adverse relational experiences during childhood, when one or more of five basic psychological needs (secure attachment, autonomy, realistic limits, self-directedness, and playfulness) are unmet [11]. When an EMS is activated, the person may respond to it by adopting a maladaptive coping style, including compensation, avoidance or surrender modalities [11]. EMS are grouped in five core domains of unmet needs; they would develop through the interactions between innate temperament and early adverse relational experiences during childhood, when one or more of five basic psychological needs (secure attachment, autonomy, realistic limits, self-directedness, and playfulness) are not satisfied by the caregivers [12]. An overview of all the EMS and the related core domains is presented in Table 1.

**Table 1:** Early maladaptive schemas and core domains

Early maladaptive schemas	Core domains
Abandonment Perceived instability or unreliability of those available for support and connection. Involves the sense that significant others will not be able to continue providing emotional support, connection, strength, or practical protection because they are emotionally unstable and unpredictable, unreliable, or erratically present; because they will die imminently; or because they will abandon the patient in favour of someone better.	Disconnection / Rejection
Mistrust / Abuse Expectation that others will hurt, abuse, humiliate, cheat, lie, manipulate, or take advantage. Usually involves the perception that the harm is intentional or the result of unjustified and extreme negligence. May include the sense that one always ends up being cheated relative to others or “getting the short end of the stick.”	
Emotional Deprivation Expectation that one’s desire for a normal degree of emotional support will not be adequately met by others. The three major forms of deprivation are: (A) Deprivation of Nurturance: Absence of attention, affection, warmth, or companionship; (B) Deprivation of Empathy: Absence of understanding, listening, self-disclosure, or mutual sharing of feelings from others; (C) Deprivation of Protection.	
Defectiveness / Shame Feeling that one is defective, bad, unwanted, inferior, or invalid in important respects; or that one would be unlovable to significant others if exposed. May involve hypersensitivity to criticism, rejection, and blame; self-consciousness, comparisons, and insecurity around others; or a sense of shame regarding one’s perceived flaws. These flaws may be private Or public.	
Social isolation / Alienation Feeling that one is isolated from the rest of the world, different from other people, and/or not part of any group or community.	
Dependence / Incompetence Belief that one is unable to handle one’s everyday responsibilities in a competent manner, without considerable help from others (e.g., take care of oneself, solve daily problems, exercise good judgment, tackle new tasks, make good decisions). Often presents as helplessness.	Impaired Autonomy
Vulnerability to harm / Illness Exaggerated fear that imminent catastrophe will strike at any time and that one will be unable to prevent it. Fears focus on one or more of the following: (A) Medical Catastrophes; (B) Emotional Catastrophes; (C) External Catastrophes.	
Enmeshment/Undeveloped self Excessive emotional involvement and closeness with one or more significant others (often parents), at the expense of full individuation or normal social development. Often involves the belief that at least one of the enmeshed individuals cannot survive or be happy without the constant support of the other.	
Failure to achieve The belief that one has failed, will inevitably fail, or is fundamentally inadequate relative to one’s peers, in areas of achievement. Often involves beliefs that one is stupid, inept, untalented, ignorant, lower in status, less successful than others.	
Entitlement / Grandiosity The belief that one is superior to other people; entitled to special rights and privileges; or not bound by the rules of reciprocity that guide normal social interaction. Often involves insistence that one should be able to do or have whatever one wants, regardless of what is realistic, what others consider reasonable, or the cost to others; or an exaggerated focus on superiority - in order to achieve power or control (not primarily for attention or approval).	Impaired Limits
Insufficient self-control / Self-discipline Pervasive difficulty or refusal to exercise sufficient self-control and frustration tolerance to achieve one’s personal goals, or to restrain the excessive expression of one’s emotions and impulses. In its milder form, patient presents with an exaggerated emphasis on discomfort-avoidance: avoiding pain, conflict, confrontation, responsibility, or overexertion---at the expense of personal fulfilment, commitment, or integrity.	
Subjugation Excessive surrendering of control to others because one feels coerced - - usually to avoid anger, retaliation, or abandonment. The two major forms of subjugation are: (A) Subjugation of Needs: Suppression of one’s preferences, decisions, and desires; (B) Subjugation of Emotions: Suppression of emotional expression, especially anger. Usually involves the perception that one’s own desires, opinions, and feelings are not valid or important to others.	Other Directedness
Self-sacrifice Excessive focus on voluntarily meeting the needs of others in daily situations, at the expense of one’s own gratification. The most common reasons are: to prevent causing pain to others; to avoid guilt from feeling selfish; or to maintain the connection with others perceived as needy. Often results from an acute sensitivity to the pain of others. Sometimes leads to a sense that one’s own needs are not being adequately met and to resentment of those who are taken care of.	
Approval-seeking / Recognition-seeking Excessive emphasis on gaining approval, recognition, or attention from other people, or fitting in, at the expense of developing a secure and true sense of self. One’s sense of esteem is dependent primarily on the reactions of others rather than on one’s own natural inclinations. Sometimes includes an overemphasis on status, appearance, social acceptance, money, or achievement -- as means of gaining approval, admiration, or attention (not primarily for power or control).	
Negativity / Pessimism A pervasive, lifelong focus on the negative aspects of life while minimizing or neglecting the positive or optimistic aspects. Usually includes an exaggerated expectation-- in a wide range of work, financial, or interpersonal situations -- that things will eventually go seriously wrong.	Overvigilance / Inhibition
Emotional inhibition The excessive inhibition of spontaneous action, feeling, or communication -- usually to avoid disapproval by others, feelings of shame, or losing control of one’s impulses. The most common areas of inhibition involve: (a) inhibition of anger & aggression; (b) inhibition of positive impulses; (c) difficulty expressing vulnerability or communicating freely about one’s feelings, needs; (d) excessive emphasis on rationality while disregarding emotions.	
Unrelenting standards / Hypercriticalness The underlying belief that one must strive to meet very high internalized standards of behaviour and performance, usually to avoid criticism. Typically results in feelings of pressure or difficulty slowing down; and in hypercriticalness toward oneself and others.	
Punitiveness The belief that people should be harshly punished for making mistakes. Involves the tendency to be angry, intolerant, punitive, and impatient with those people (including oneself) who do not meet one’s expectations or standards. Usually includes difficulty forgiving mistakes in oneself or others, because of a reluctance to consider extenuating circumstances, allow for human imperfection, or empathize with feelings.	

EMS often act outside of cognitive awareness as a vulnerability factor for a variety of psychological conditions, including depressive/anxiety and personality disorders [11, 13–15]. Schema Therapy (ST) [11], developed to modify EMS, assumes that childhood experiences have a key role in the development of emotion regulation strategies. Adverse childhood experiences can lead to fear and avoidance of negative emotions [11].

Cognitive behavioural models explained development and maintenance processes of SP to help conceptualization in clinical practice [16]. SP behaviours are triggered by biased thoughts/beliefs deriving from EMS (“*People won’t like me at the party*”); activation of such thoughts leads to negative emotions, then to enactment of SP behaviour. Positive/negative reinforcement plays as maintenance process. However, no study examined the role of EMS in SP behaviours and subtypes.

### Rationale and objectives

It is important to understand processes associated with SP to refine its conceptualization, then optimise treatment. The role of personality is under-investigated: no study examined EMS across subtypes. Understanding which EMS are associated with subtypes may suggest the future use of ST according to the SP subtype. The current study explored the relationship between EMS and SP subtypes in community adults who self-reported SP behaviours.

## Material and methods

### Participants

Five-hundred ninety-six community adults (Table 2) [mean age = 35.23 years, 66% females] were recruited through convenience sampling by psychologists in different public settings (universities, libraries, sports/volunteering associations). Participants were identified in the context of a large survey on body focused repetitive behaviours conducted in the general population. Participants were provided with study aims, a description of SP behaviours, the fact that they occur in the general population. Eligibility criteria included the fact that participants had provided written informed consent and that they had scored at least one standard deviation above the normative mean [6] on the Milwaukee Inventory for the Dimensions of Adult Skin Picking (MIDAS) [5], a self-report measure of SP behaviours, suggesting the presence of SP behaviours to some extent. Participation was anonymous, voluntary and uncompensated. The study was approved by the ethical committee of the university institution where it was conducted.

**Table 2:** Demographics of the group (*n* = 596)

	M (SD; range)	n (%)
Age (years)	35.23 (13.79; 18–76)	
Females		397 (66)
Marital status		
Single		369 (62)
Married		156 (26.2)
Separated/divorced		61 (10.2)
Widowed		10 (1.7)
Occupation		
Student		194 (32.5)
Working		248 (41.6)
Unemployed		22 (3.7)
Retired		31 (5.2)
Education		
Elementary-school		8 (1.3)
Middle-school		104 (17.4)
High-school		230 (38.6)
Degree		190 (31.9)
Ph.D/specialization		64 (11.7)

### Measures

When each participant was considered as eligible, he/she was taken aside to complete two questionnaires individually: the MIDAS [5] and the Young Schema Questionnaire-Long form third version (YSQ-L3) [17].

The MIDAS is a 12-item questionnaire assessing SP subtypes: each item is rated from 1 (not true for any of my SP behaviours) to 5 (true for all my SP behaviours). It is the only measure of SP subtypes: a Focused one, which concerns specific body areas and occurs in response to negative emotions or bodily sensations, and an Automatic subtype, which occurs without awareness during activities not related to picking. On 92 participants self-reporting SP, the validation study [5] identified two subscales, measuring respectively Focused and Automatic SP. The Italian version [6] showed 3 factors, Focused (example item: “I pick my skin when I am experiencing a negative emotion such as stress, anger, frustration, or sadness”), Automatic (“I pick my skin when I am concentrating on another activity”), and Mixed SP (“I pick my skin while I am looking in the mirror”).^13^ In the current group, internal consistency was good for Focused (Cronbach’s alpha = 0.87) and Automatic (Cronbach’s alpha = 0.81) and acceptable for the Mixed scale (Cronbach’s alpha = 0.70).

The YSQ-L3 is a 232-item questionnaire assessing 18 EMS. Participants are asked to rate each statement on a 6-point Likert scale (not true at all = 1, this describes me perfectly = 6). Items are clustered by 18 scales and grouped into 5 domains, bringing together the EMS that develop together: (1) Disconnection/Rejection (EMS: Abandonment, Mistrust/Abuse, Emotional Deprivation, Defectiveness/Shame, Social Isolation/Alienation); (2) Impaired Autonomy/Performance (EMS: Dependence/Incompetence, Vulnerability to Harm/Illness, Enmeshment/Undeveloped Self, Failure); (3) Impaired Limits (EMS: Entitlement/Grandiosity, Insufficient Self-Control/Self-Discipline); (4) Other-Directedness (EMS: Subjugation, Self-Sacrifice, Approval/Recognition-Seeking); and (5) Overvigilance/Inhibition (EMS: Negativity/Pessimism, Emotional Inhibition, Unrelenting Standards/Hypercriticalness, Punitiveness). Higher scores reflect stronger EMS. In the current group, internal consistency was good to excellent across the 18 scales (Cronbach’s alpha range = 0.82–0.91).

### Statistical analysis

To test the relationship between SP subtypes and EMS, Pearson’s bivariate correlation coefficients were calculated between MIDAS and YSQ-L3 scores. Coefficient values were interpreted as: 0 < *r* < |0.30| = weak, |0.30| < *r* < |0.50| = moderate, |0.50| < *r* < ±|0.70| = strong, *r* < ±|0.70| = very strong. Fisher’s *z* coefficients were computed to compare the strength of the correlation indices between the scores on each one of the MIDAS scales and the scores on each one of the YSQ-L3 scales. Analyses of variance were carried out to explore gender differences on MIDAS scores. Analyses of covariance were conducted by including YSQ-L3 scale scores, age and gender as predictors and MIDAS scores (Focused, Automatic, Mixed scores) as outcomes. Statistical analyses were conducted through SPSS version 25.0 with a significance level set at *p* < 0.05.

## Results

### Correlations between EMS and SP subtypes

Pearson’s coefficients between MIDAS and YSQ-L3 scores are presented in Table 3. MIDAS Automatic scores correlated positively and strongly with MIDAS Focused scores and positively and moderately with MIDAS Mixed scores. MIDAS Focused scores correlated moderately and positively with MIDAS Mixed scores.

**Table 3:** Bivariate correlations between MIDAS and YSQ-L3 scores and gender differences on MIDAS scores (*n* = 596)

	Pearson’s bivariate correlation coefficients						
	MIDAS Automatic		MIDAS Focused		MIDAS Mixed		Fisher’s *z* (*p*-value)
MIDAS Automatic							
MIDAS Focused	0.67^**^						
MIDAS Mixed	0.42^**^		0.42^**^				
YSQ-L3 Emotional Deprivation	−0.20^**^		−0.17^**^		−0.16^**^		0.77 (0.219)
YSQ-L3 Abandonment	0.21^**^		0.23^**^		0.26^**^		1.02 (0.154)
YSQ-L3 Mistrust/Abuse	0.17^**^		0.22^**^		0.22^**^		0.00 (0.500)
YSQ-L3 Social Isolation/Alienation	0.16^**^		0.10^*^		0.24^**^		2.69 (0.004)
YSQ-L3 Defectiveness/Shame	0.21^**^		0.18^**^		0.27^**^		1.81 (0.035)
YSQ-L3 Failure to Achieve	0.22^**^		0.19^**^		0.30^**^		2.24 (0.012)
YSQ-L3 Dependence/Incompetence	0.30^**^		0.24^**^		0.22^**^		1.66 (0.048)
YSQ-L3 Vulnerability to Harm/Illness	0.21^**^		0.24^**^		0.25^**^		0.82 (0.206)
YSQ-L3 Enmeshment/Undeveloped Self	0.17^**^		0.16^**^		0.17^**^		0.19 (0.424)
YSQ-L3 Subjugation	0.21^**^		0.19^**^		0.20^**^		0.39 (0.346)
YSQ-L3 Self-sacrifice	0.12^**^		0.15^**^		0.19^**^		0.75 (0.227)
YSQ-L3 Emotional Inhibition	0.23^**^		0.18^**^		0.22^**^		1.00 (0.157)
YSQ-L3 Unrelenting Standard/Hypercriticalness	0.22^**^		0.22^**^		0.18^**^		0 (0.500)
YSQ-L3 Entitlement/Grandiosity	0.21^**^		0.20^**^		0.18^**^		0.19 (4.422)
YSQ-L3 Insufficient Self-Control/Self-Discipline	0.22^**^		0.20^**^		0.26^**^		0.41 (0.340)
YSQ-L3 Approval/Recognition Seeking	0.30^**^		0.22^**^		0.22^**^		1.64 (0.051)
YSQ-L3 Negativity/Pessimism	0.22^**^		0.24^**^		0.30^**^		1.66 (0.048)
YSQ-L3 Punitiveness	0.23^**^		0.23^**^		0.26^**^		0 (0.500)
Age	−0.25^**^		−0.25^**^		− 0.22^**^		
Gender	MIDAS Automatic		MIDAS Focused		MIDAS Mixed		
	Mean *(SD)*	*F*_*(1, 594)*_	Mean (*SD)*	*F*_*(1, 594)*_	Mean *(SD)*	*F*_*(1, 594)*_	
Females	8.51 (4.27)	0.01	5.75 (3.27)	1.06	6.47 (2.68)	4.56*	
Males	8.49 (4.54)		5.46 (3.19)		5.93 (3.02)		

*YSQ – L3* Young Schema Questionnaire – L3 version, *MIDAS* Milwaukee Inventory for the Dimensions of Adult Skin Picking

** *p* < 0.01, * *p* < 0.05

MIDAS Automatic scores correlated positively and weakly with scores on all the YSQ-L3 scales, except with YSQ-L3 Approval/Recognition Seeking and YSQ-L3 Dependence/Incompetence scores, which correlated moderately with MIDAS Automatic scores. MIDAS Automatic scores correlated negatively and weakly with YSQ-L3 Emotional Deprivation scores.

MIDAS Focused scores correlated positively and weakly with all YSQ-L3 scale scores and negatively and weakly with YSQ-L3 Emotional Deprivation scores.

MIDAS Mixed scores correlated positively and moderately with YSQ-L3 Failure to Achieve and with YSQ-L3 Negativity/Pessimism scores and positively and weakly with the scores on all the other YSQ-L3 scales, except for YSQ-L3 Emotional Deprivation, which correlated weakly and negatively with MIDAS Mixed scores.

Age correlated negatively and weakly with all the MIDAS scales scores: the intensity of all the three SP subtypes was higher for younger individuals. Gender differences were found only on MIDAS Mixed scores: females had significantly higher scores than males.

The results of Fisher’s *z* tests showed that scores on the MIDAS Mixed scale correlated with scores on the YSQ-L3 Social Isolation/Alienation, YSQ-L3 Defectiveness/Shame, YSQ-L3 Failure to Achieve, and YSQ-L3 Negativity/Pessimism scales more strongly than scores on the other MIDAS scales. Scores on the MIDAS Automatic scale correlated with scores on the YSQ-L3 Dependence/Incompetence scale more strongly than scores on the other MIDAS scales.

### Predictive effect of EMS on SP subtypes

The results of analyses of covariance are in Tables 4, 5 and 6. YSQ-L3 scores, age (which correlated negatively and weakly with all the MIDAS scale scores) and gender were included as predictors and MIDAS scores as outcomes. Higher YSQ-L3 Dependence/Incompetence and YSQ-L3 Approval/Recognition Seeking scores predicted higher MIDAS Automatic scores, while lower YSQ-L3 Emotional Deprivation scores and younger age predicted higher MIDAS Automatic scores. Scores on the other scales of the YSQ-L3 did not predict significantly MIDAS Automatic scores.

**Table 4:** Multiple linear regression of MIDAS automatic scores on YSQ – L3 scores (*n* = 596)

Predictors	***B***	***t***	***P***-value	95% confidence interval		Partial ***η***^***2***^
				Lower limit	Upper limit	
Intercept	6780	7457	,000	4994	8565	,088
Male gender	-,578	− 1581	,114	− 1295	,140	,004
Female gender	0^a^	.	.	.	.	.
Age (years)	-,055	− 4116	< 0.001	-,081	-,029	,029
YSQ-L3 Emotional Deprivation	-,006	− 3812	< 0.001	-,010	-,003	,025
YSQ-L3 Abandonment	,014	,776	,438	-,021	,049	,001
YSQ-L3 Mistrust/Abuse	-,002	-,088	,930	-,041	,037	,000
YSQ-L3 Social Isolation/Alienation	-,047	− 1440	,150	-,111	,017	,004
YSQ-L3 Defectiveness/Shame	,044	1396	,163	-,018	,107	,003
YSQ-L3 Failure to Achieve	-,028	-,776	,438	-,100	,043	,001
YSQ-L3 Dependence/Incompetence	,084	2731	,007	,024	,145	,013
YSQ-L3 Vulnerability to Harm/Illness	,029	1039	,299	-,025	,082	,002
YSQ-L3 Enmeshment/Undeveloped Self	-,057	− 1797	,073	-,119	,005	,006
YSQ-L3 Subjugation	−0.001	-,001	,999	-,069	,069	,000
YSQ-L3 Self-sacrifice	-,011	-,707	,480	-,043	,020	,001
YSQ-L3 Emotional Inhibition	,030	,893	,372	-,036	,095	,001
YSQ-L3 Unrelenting Standard/Hypercriticalness	,034	1793	,073	-,003	,071	,006
YSQ-L3 Entitlement/Grandiosity	-,004	-,133	,895	-,066	,057	,000
YSQ-L3 Insufficient Self-Control/Self-Discipline	-,008	-,339	,734	-,056	,040	,000
YSQ-L3 Approval/Recognition Seeking	,049	2026	,043	,001	,096	,007
YSQ-L3 Negativity/Pessimism	-,026	-,996	,320	-,079	,026	,002
YSQ-L3 Punitiveness	,028	1192	,234	-,018	,073	,002

*YSQ - L3* Young Schema Questionnaire – Long form third version, *MIDAS* Milwaukee Inventory for the Dimensions of Adult Skin Picking

^a^Parameter set at 0 because redundant

**Table 5:** Multiple linear regression of MIDAS focused scores on YSQ - L3 scores (*n* = 596)

Predictors	***B***	***t***	***P***-value	95% confidence interval		Partial ***η***^***2***^
				Lower limit	Upper limit	
Intercept	4433	6542	< 0.001	3102	5764	,069
Male gender	-,460	− 1690	,092	-,995	,075	,005
Female gender	0^a^	.	.	.	.	.
Age (years)	-,042	− 4258	< 0.001	-,061	-,023	,031
YSQ-L3 Emotional Deprivation	-,004	− 3155	,002	-,007	-,002	,017
YSQ-L3 Abandonment	,013	,946	,344	-,014	,039	,002
YSQ-L3 Mistrust/Abuse	,033	2197	,028	,003	,062	,008
YSQ-L3 Social Isolation/Alienation	-,085	− 3508	< 0.001	-,133	-,038	,021
YSQ-L3 Defectiveness/Shame	,034	1432	,153	-,013	,080	,004
YSQ-L3 Failure to Achieve	-,007	-,256	,798	-,060	,046	,000
YSQ-L3 Dependence/Incompetence	,052	2267	,024	,007	,098	,009
YSQ-L3 Vulnerability to Harm/Illness	,033	1621	,106	-,007	,073	,005
YSQ-L3 Enmeshment/Undeveloped Self	-,052	− 2192	,029	-,098	-,005	,008
YSQ-L3 Subjugation	,015	,562	,575	-,037	,066	,001
YSQ-L3 Self-sacrifice	,001	,101	,920	-,022	,025	,000
YSQ-L3 Emotional Inhibition	-,028	− 1121	,263	-,076	,021	,002
YSQ-L3 Unrelenting Standard/Hypercriticalness	,019	1335	,182	-,009	,047	,003
YSQ-L3 Entitlement/Grandiosity	-,004	-,168	,867	-,050	,042	,000
YSQ-L3 Insufficient Self-Control/Self-Discipline	-,008	-,416	,677	-,043	,028	,000
YSQ-L3 Approval/Recognition Seeking	,009	,505	,614	-,026	,044	,000
YSQ-L3 Negativity/Pessimism	,002	,105	,916	-,037	,041	,000
YSQ-L3 Punitiveness	,023	1314	,189	-,011	,057	,003

*YSQ - L3* Young Schema Questionnaire – Long form third version, *MIDAS* Milwaukee Inventory for the Dimensions of Adult Skin Picking

^a^Parameter set at 0 because redundant

**Table 6:** Multiple linear regression of MIDAS Mixed scores on YSQ-L3 scores (*n* = 596)

Predictors	***B***	***t***	***P***-value	95% confidence interval		Partial ***η***^***2***^
				Lower limit	Upper limit	
Intercept	4886	7967	< 0.001	3681	6090	,099
Male gender	-,579	− 2349	,019	− 1063	-,095	,010
Female gender	0^a^	.	.	.	.	.
Age (years)	-,027	− 3024	,003	-,045	-,009	,016
YSQ-L3 Emotional Deprivation	-,005	− 4025	< 0.001	-,007	-,002	,027
YSQ-L3 Abandonment	,009	,782	,434	-,014	,033	,001
YSQ-L3 Mistrust/Abuse	,002	,171	,864	-,024	,029	,000
YSQ-L3 Social Isolation/Alienation	,009	,426	,670	-,034	,053	,000
YSQ-L3 Defectiveness/Shame	,029	1330	,184	-,014	,071	,003
YSQ-L3 Failure to Achieve	,048	1949	,052	,000	,096	,007
YSQ-L3 Dependence/Incompetence	-,026	− 1268	,205	-,067	,015	,003
YSQ-L3 Vulnerability to Harm/Illness	,030	1603	,109	-,007	,066	,004
YSQ-L3 Enmeshment/Undeveloped Self	-,025	− 1175	,240	-,067	,017	,002
YSQ-L3 Subjugation	-,014	-,586	,558	-,060	,033	,001
YSQ-L3 Self-sacrifice	,014	1337	,182	-,007	,036	,003
YSQ-L3 Emotional Inhibition	-,018	-,809	,419	-,062	,026	,001
YSQ-L3 Unrelenting Standard/Hypercriticalness	-,005	-,413	,680	-,030	,020	,000
YSQ-L3 Entitlement/Grandiosity	-,017	-,805	,421	-,059	,024	,001
YSQ-L3 Insufficient Self-Control/Self-Discipline	,027	1619	,106	-,006	,059	,005
YSQ-L3 Approval/Recognition Seeking	,008	,507	,612	-,024	,040	,000
YSQ-L3 Negativity/Pessimism	,007	,417	,677	-,028	,043	,000
YSQ-L3 Punitiveness	,021	1350	,178	-,010	,052	,003

*YSQ - L3* Young Schema Questionnaire – Long form third version, *MIDAS* Milwaukee Inventory for the Dimensions of Adult Skin Picking

^a^Parameter set at 0 because redundant

Higher YSQ-L3 Dependence/Incompetence and YSQ-L3 Mistrust/Abuse scores predicted higher MIDAS Focused scores. Lower YSQ-L3 Emotional Deprivation, YSQ-L3 Social Isolation/Alienation, YSQ-L3 Enmeshment/Undeveloped Self scores and younger age predicted higher MIDAS Focused scores. Scores on the other scales of the YSQ-L3 did not predict significantly MIDAS Focused scores.

Lower YSQ-L3 Emotional Deprivation scores, male gender and younger age predicted higher MIDAS Mixed scores. Higher YSQ-L3 Failure to Achieve scores predicted higher MIDAS Mixed scores at borderline significance. Scores on the other scales of the YSQ-L3 did not predict MIDAS Mixed scores.

In conclusion, higher Dependence/Incompetence EMS was a common predictor of both Focused and Automatic subtypes, while lower Emotional Deprivation EMS and age predicted all three subtypes. Higher Approval/Recognition Seeking, higher Mistrust/Abuse and higher Failure to Achieve were specific predictors of Automatic, Focused and Mixed subtypes, respectively. Lower Social Isolation/Alienation and Enmeshment/Undeveloped Self were specific predictors of Focused subtype. Male gender was a specific predictor of Mixed subtype.

## Discussion

Little research investigated personality features in SP subtypes. The present study was the first contribution assessing the role of EMS in SP subtypes in a large community group self-reporting picking behaviour. Understanding processes associated with SP might help development of tailored conceptualization and treatment strategies targeting different EMS for different subtypes.

Intercorrelation between Automatic and Focused subtypes was strong, whereas correlations of both Automatic and Focused subtypes with Mixed one were moderate. This finding was in contrast with evidence [5] showing that the subtypes were not correlated but it was consistent with prior research reporting moderate to strong intercorrelations [6].

Correlations between subtypes and EMS were all positive except for Emotional Deprivation EMS, which negatively correlated with all three subtypes: individuals with higher expectation that their needs for emotional support will not be adequately met by others reported SP to less extent than those reporting stronger Emotional Deprivation EMS.

Correlations between three SP subtypes and EMS were all weak, except for the association between Mixed SP and Negativity/Pessimism and Failure to Achieve EMS, which were moderate. Thus, individuals self-reporting Mixed SP endorsed stronger beliefs of being fundamentally inadequate relative to peers in areas of achievement and a focus on the negative aspects of life while neglecting the positive ones. This evidence is consistent with the association between Mixed SP and depression [6]. A moderate correlation was found also between Automatic SP and Approval/Recognition Seeking EMS: individuals with more intense Automatic SP behaviour tend to put excessive emphasis on gaining approval from others, at the expense of developing a true sense of self, consistently with reports indicating approval seeking and separation anxiety as predictors of avoidant personality [18]. This evidence might support the hypothesis that avoidant personality traits might be involved in the Automatic subtype, and that this subtype might be related to the so-called vulnerable narcissistic personality picture, often associated to avoidant personality, in the same manner as self-injurious behaviour [19]. Analyses of covariance showed that the three SP subtypes were associated with common EMS and differentially with specific EMS.

### EMS common to subtypes

Higher Dependence/Incompetence EMS predicted stronger both Automatic and Focused SP, whereas lower Emotional Deprivation was common to all subtypes. This result indicated that individuals viewing themselves as unable to handle everyday responsibilities without help from others tend to report both subtypes. The relation between the two subtypes and Dependence/Incompetence EMS may be explained by the fact that individuals who are not able to stop picking may feel frustrated, then may get into a helpless vicious cycle [20]. The connection between these EMS and SP might be an effect also of the association between subtypes, particularly Focused, and depression [21]. This result is consistent with a study [22] showing that Dependence/Incompetence EMS were associated with non-suicidal self-injurious behaviour. Supporting the link between Emotional Dependence and SP, Estévez and colleagues [23] reported that Emotional Dependence was associated with impulsive behaviour and that it mediated the relation between Attachment and Impulsivity. The result about the role of Dependence in Automatic SP is interesting since loneliness was an emotional trigger of SP behaviour [24]. In addition, the association between a sense of incompetence and SP may be attributed to a negative self-judgemental attitude, a cognitive factor found to be related to depressive traits [25].

Emotional Deprivation EMS resulted a protective factor against all the three subtypes of SP, as more intense SP behaviour was associated with lower Emotional Deprivation. This result indicated that those individuals self-reporting picking behaviour to less extent believed that their needs for emotional support are not adequately met by others. This result might be in contrast with the literature, since Emotional Deprivation is believed a risk or maintenance factor for a number of symptoms and disorders [26]. However, it might be hypothesized that individuals who have SP tendencies have difficulties identifying/describing feelings, given the relation between Alexithymia and SP [21]; thus, they could not identify these needs as unmet. Since Obsessive Compulsive Disorder (OCD) and body focused repetitive behaviours have clinical overlap [20], a further explanation might be in research on parenting associated with development of OCD [27], where OCD severity was linked with parental overprotection.

Younger age predicted stronger levels on all three SP subtypes, supporting evidence that SP behaviours concern mostly late adolescents and young adults [28].

### EMS specific to SP subtypes

Higher Approval/Recognition Seeking predicted stronger Automatic SP. This association might be explained by perfectionism reported in SP: feelings of dissatisfaction and imperfection regarding the body can activate the picking episode with the aim to obtain approval/recognition by others and avoid feelings of shame or social rejection [28, 29]. Many individuals suffering from SP begin picking at an area of imperfection/blemish that they fixate on [30]. The role of Approval/Recognition Seeking seemed in line with case reports of Body Focused Repetitive Behaviours: Pélissier and O’Connor [31] described a patient who identified frustration/impatience as dominant emotions during the injurious behaviour (occurring when she was waiting or on wasting time) and identified automatic thoughts (“I’m not fast enough”, “I’m not performing well”), which increased her tension provoking the behaviour. Approval/Recognition Seeking might be considered an outcome of an authoritarian parenting and less parental acceptance which literature found playing a role in the development of OCD spectrum [27, 32].

Higher Emotional Deprivation predicted less intense both Automatic and Focused SP. This result was in contrast with previous evidence [9], where avoidant traits were related to SP and with research [26] indicating that shame proneness/social avoidance were associated with SP, due to skin lesions. Maybe the use of a community group accounts for this difference, as the individuals did not have clinical SP resulting in tissue damage.

Higher Mistrust/Abuse EMS predicted Focused subtype. This relation was consistent with evidence demonstrating that the Focused subtype is associated with borderline traits [10], since the Disconnection/Rejection domain was the most specific to borderline personality and Abuse/Mistrust EMS was one of the most closely associated EMS to borderline traits [26]. The association between Focused SP and borderline personality was demonstrated in a study where patients with Borderline Personality Disorder reported higher Focused SP behaviour than controls [10]. The role of Mistrust/Abuse EMS may be consistent with evidence showing that SP is associated with a history of childhood sexual/interpersonal abuse [33], which often predicts borderline personality [34], The association between Mistrust/Abuse EMS and Focused SP found in the current study might be consistent with models of self-injurious behaviour, which is seen as a compensatory regulation in posttraumatic adaptation [35]. In a study with outpatients, Mistrust/Abuse EMS correlated moderately with socially avoidant and vindictive interpersonal behaviours. This evidence supports the relation found in the current study between Mistrust/Abuse and SP, as in previous research also avoidant and passive–aggressive personality predicted significantly the Focused SP subtype [10].

The lack of a significant relation between SP subtypes and Shame/Defectiveness EMS appeared in contrast with studies demonstrating an association between social avoidance/shame and SP behaviour [36].

Higher Failure to Achieve EMS predicted stronger Mixed SP tendencies. Individuals with stronger Mixed SP tendencies seem to focus on the negative aspects of life while minimizing the positive ones and viewing themselves as inadequate relative to peers. These associations might be explained by the role of negative self-evaluation including higher disgust propensity in OCD spectrum disorders [37, 38].

## Conclusions

The three SP subtypes were associated with common EMS and with distinct EMS. Higher Dependence/Incompetence EMS was a common predictor of both Focused and Automatic subtypes, while lower Emotional Deprivation EMS and age were common predictors of all three subtypes. Higher Approval/Recognition Seeking, higher Mistrust/Abuse and higher Failure to Achieve were specific predictors of Automatic, Focused and Mixed subtypes, respectively. Lower Social Isolation/Alienation and Enmeshment/Undeveloped Self were specific predictors of Focused subtype. Male gender was a specific predictor of Mixed subtype.

Some limitations should be considered. First, the study used a community group reporting SP but it did not ascertain the diagnosis of SP through a clinician-administered instrument. The cross-sectional design prevented conclusions about the causal link between EMS and SP: EMS might be reinforced by the consequences produced by picking behaviour in the body and the difficulty stopping it (e.g., Dependence/Incompetence EMS). A longitudinal design would provide more confidence in the associations among these variables, since the variables are measured at two (or more) points in time, with lag relationships assessed.

The effects of some clinical variables, such as anxiety and depression, were not controlled for. Given that the study consists of correlating one set of self-report scales that involve reporting negative aspects about oneself (i.e., skin picking behaviours) with another set of scales that involve reporting negative aspects about oneself (i.e., EMS), a key point would be to control for the large component of self-report scales related to negative reporting about oneself. It might be expected that most of correlations found would reduce considerably once this reporting bias is controlled. Alternative methods to measure the activation of EMS as triggers of SP episodes might be useful. Future studies for example might use app-based diaries to assess whether activation of EMS precedes picking.

In conclusion, the present findings expand the literature on the relationship between personality characteristics and SP subtypes, investigating for the first time the role of EMS: SP subtypes seem to be associated with common and specific EMS. This highlights a potential future application and evaluation of ST in a personalised approach for this impairing, yet under-investigated, condition.

## Data Availability

The datasets used and/or analysed during the current study are available from the corresponding author on reasonable request.

## Abbreviations

EMS: Early Maladaptive Schemas
MIDAS: Milwaukee Inventory for the Dimensions of Adult Skin Picking
OCD: Obsessive-Compulsive Disorder
SP: Skin Picking
ST: Schema Therapy
YSQ-L3: Young Schema Questionnaire-Long form third version
